# Co-existence of superficial brachio-ulno-radial arterial pattern and persistent median artery

**DOI:** 10.4103/0970-0358.53021

**Published:** 2009

**Authors:** Lakshmi Ramanathan, Soubhagya R. Nayak, K. V. Vinay, Ashwin Krishnamurthy, Latha V. Prabhu

**Affiliations:** Department of Anatomy, Centre for Basic Sciences, Kasturba Medical College, Bejai, Mangalore - 575 004, Karnataka, India

**Keywords:** Median artery, bnormal arterial pattern, Superficial arteries of upper limb

## Abstract

The arteries of the upper limbs are situated in a deep plane and are the favourable sites for intra-arterial cannulation. During routine dissection of the left upper limb of a 52-year-old female cadaver, we observed a superficial arterial pattern which was of superficial brachio-ulno-radial type. The right upper limb of the same individual did not show any abnormal arterial pattern. This superficial arterial system was also associated with a palmar type of median artery. The clinical significance of the anomalous arterial system of the upper limb is discussed.

## INTRODUCTION

Superficial brachio-ulno-radial (SBUR) type is defined as superficial brachial artery branching at the elbow level into the radial and ulnar arteries coursing over the superficial forearm flexors, and coexisting in the whole arterial pattern of the limb with a normal brachial artery that continues as the common interosseous trunk. The occurrence of the SBUR in human embryos was found to be 0.7%.[1] The incidence of SBUR in adults has been found ranging from 0.14-1.3%.[2–5] Rodriguez-Niedenfuhr *et al.*, found the above arterial pattern in two of 192 cadavers (1.04%).[6] In the present case the SBUR was also associated with a palmar type of median artery. The clinical significance of the present variation is discussed.

## CASE REPORT

**Figure 1:**
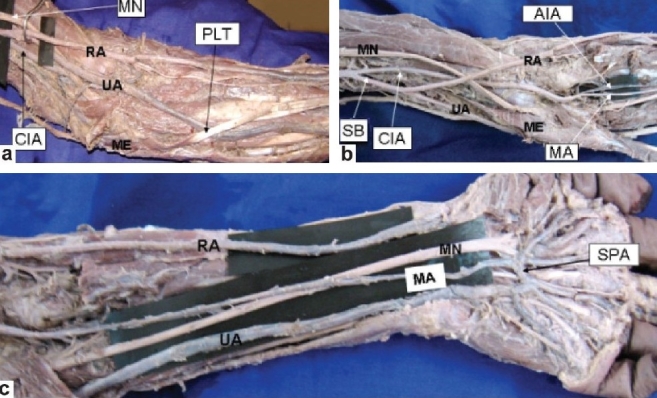
Ventral aspect of the left upper limb (a) CIA – Common interosseous artery; ME – medial epicondyle; MN – median nerve; PLT – palmaris longus tendon; RA – radial artery; UA – ulnar artery. Note the PLT is crossing over the UA (b) AIA – Anterior interosseous artery; CIA – Common interosseous artery; MA – Median artery; ME – Medial epicondyle; MN – Median nerve; RA – Radial artery; SB – Superficial brachial; UA – Ulnar artery (c) MA – Median artery; MN – Median nerve; RA – Radial artery; SPA – Superficial palmar arch; UA – Ulnar artery

During routine dissection of the left upper limb of a 52-year-old female cadaver, we observed that the limb presented a superficial arterial pattern which was of SBUR type. This superficial arterial system was also associated with a palmar type of median artery. The brachial artery presented as the continuation of the axillary artery and was found to give a common interosseous branch at about 10.5 cm above the intercondylar line [Figure 1b]. Thereafter the superficial brachial artery (SBA) which ran superficial to the muscles of the arm went on to divide into superficial radial (SR) and superficial ulnar (SU) arteries, 7 cm above the intercondylar line [Figure 1b]. Both these branches ran superficial to the forearm flexors except the palmaris longus (PL), which was crossing the SU artery [Figure 1a]. The SU artery crossed the median nerve superficial to it from the lateral to the medial side and terminated by forming the superficial palmar arch (SPA), which was completed by the median artery to form an arch of medio-ulnar type [Figure 1c]. The common interosseous artery (CIA) passed laterally and ran deep to the median nerve at the middle of the arm and emerged between the median nerve and the bicipital tendon at the cubital fossa. The artery then ran deep to the pronator teres and trifurcated at the level of the neck of the radius into the median artery, anterior interosseous artery and the posterior interosseous artery [Figure 1b]. The median artery then ran along with the median nerve and crossed it in the distal part of the forearm to enter the palm below the flexor retinaculum and went on to complete the SPA along with the SU artery [Figure 1c]. The right upper limb of the same individual did not show any abnormal arterial pattern.

## DISCUSSION

The upper limb arteries develop in five stages.[13] An axial arterial pattern represented in the adult by axillary artery, brachial artery and interosseous artery develops first while other branches develop later from the axial system. In the later stages the median artery branches from the anterior interosseous artery and the ulnar artery branches from the brachial artery respectively. In the further course of development a superficial brachial artery arises from the axillary artery and it continues as radial artery. Regression of the median artery and an anastomosis between the brachial artery and superficial brachial artery with regression of the proximal segment of the latter gives rise to the definitive radial artery. The present anomaly can be explained by the persistence of embryological vessels, which may be due to haemodynamic persistence of superficial system over deep system at the origin of superficial brachial artery. Genetic influences are deemed to be prevalent causes of such variation, although factors like foetal position *in utero*, first limb movement or unusual musculature cannot be excluded.

Generally, arteries are situated in a deep plane in the upper limb making it a favourable site for intra-arterial cannulation, but at times the presence of a superficial arterial system may cause havoc to unsuspecting surgeons leading to various complications like inadvertent intra-arterial cannulation.[7] Incidence of such a superficial arterial system of the upper limb may be of different types depending on the arteries involved. In the present case we found a superficial arterial pattern of brachial, ulnar and radial arteries. This type of arterial pattern was termed as superficial brachioulno-radial (SBUR) by Moncayo-Marques.[8]

D'costa *et al*., have reported a case of SBUR artery wherein the brachial artery was superficial and divided into ulnar and radial arteries, the CIA being a branch from the superficial radial artery originating 1 cm distal to the bifurcation of SBA. In the case being reported, however, the CIA was a branch of the brachial artery.[9] A normal brachial artery usually accompanies SBUR,[10] although this was not observed in the present case. The presence of SBUR type of arterial pattern may be of immense clinical significance since its presence can facilitate a skin flap which can be used for various plastic and reconstructive surgeries.[11] The existence of a superficial radial artery implies the absence of the normal radial pulse at wrist level and may produce problems in cannulation for per operative monitoring.[12] The crossing of PL tendon over the SU artery in the present case should be kept in mind while performing PL tendon transfer or tendon harvest and while dissecting in and around the cubital fossa.

Variation in the branching pattern of the brachial artery is of significance in cardiac catheterization for angioplasty, pedicle flaps, or arterial grafting.[14] Sajja *et al.*, described that the radial artery is suitable for coronary artery bypass grafting and is growing in popularity among cardiac surgeons.[15] But when there is a superficial arterial pattern present in the arm and forearm as in the present case, it becomes more vulnerable to trauma and thus to haemorrhage but, at same time, more accessible for cannulation, if necessary. If they are superficial to flexor muscles, the radial and ulnar artery may be mistaken for veins. Such misinterpretations can lead to intra-arterial injections, wrong interpretations of incomplete angiographic images or severe disturbances of hand irrigation during surgical procedures on the arm or forearm. [16]

Vascular anomalies occurring in common surgical sites (arm and forearm) as in the present case tend to increase the likelihood of damaging the superficial anomalous arteries during bypass graft surgery and plastic surgery. Thus it is important for surgeons and radiologists to be aware of the possible arterial variations of SBUR, in order to prevent complications during surgical and diagnostic procedures. The present variation is of some interest to hand surgeons too, as the existence of such a branching pattern can be accidently encountered while harvesting flaps from the forearm. One should also suspect the anomaly if the ulnar/radial pulse is not palpable at the wrist as usual. It is also important for the reconstructive surgeons to have knowledge of the present anomalous arterial pattern while performing skin flaps so that accidental devascularization of the distal extremity does not occur after free flap harvest. The additional presence of medio-ulnar type of SPA with SBUR in the present case makes it more interesting and clinically significant.
